# Colorectal Carcinoma Screening in Lagos, Nigeria, Are We Doing it Right?

**DOI:** 10.4021/gr2009.01.1256

**Published:** 2009-01-20

**Authors:** Charles A Onyekwere, Anthonia O Ogbera, Fatima B Abdulkareem, John Ashindoitiang

**Affiliations:** a Department of Internal Medicine, Lagos State University Teaching Hospital, Ikeja, Lagos, Nigeria; b Department of Morbid Anatomy, College of Medicine University of Lagos; c Department of Surgery, Motayo Hospital, Ikeja

**Keywords:** Colorectal cancer, Screening, Nigeria

## Abstract

**Background:**

Screening for colorectal cancer (CRC) has proven effective in reducing disease mortality and is also cost effective. Recent reports indicate that colorectal cancer is not uncommon and presents with advanced disease in Nigeria. Thus this study was aimed at reviewing the practice of CRC screening among medical practitioners in Nigeria.

**Methods:**

A self-administered questionnaire was utilized to obtain data for this study, which was distributed to over 500 practising doctors in Lagos, Nigeria from September to November 2007. The data obtained from the questionnaire include basic demographics, type of practice, duration in years of medical practice described as short (≤ 5 years), medium (5 to 10 years) or long (> 10 years), and knowledge regarding CRC, as well as CRC screening techniques and methodologies.

**Results:**

There were 300 respondents with a mean age (SD) of 33 (7.8) years and an age range of 23 - 67 years. In terms of duration of medical practice, 190 (63%) were short, 43 (14%) medium and 67 (23%) long. Majority (65%) of the respondents were in teaching hospitals, 18.5% in private hospitals and 5.7% were in general (community) hospitals. The knowledge of the clinical features as well as the risk factors of CRC was fair in over 75% of the respondents. Most respondents, 265 (87.8%), agreed that CRC was worth screening for; 21 (5%) did not. In all, 246 (82%) gave reasons for their responses. However, just over half of the respondents employed one of the following: faecal occult blood test (FOBT), double contrast barium enema (DCBE), flexible sigmoidoscopy, colonoscopy, or a combination of any of the techniques for screening. Usage of CT colonography was low. Screening rates by respondents for other malignancies in this survey was higher than that of CRC (prostate 95%, breast 97%, cervix 99%), though the most commonly encountered malignancy was breast cancer. On the contrary, for surveillance purposes, barely half of the respondents used FOBT annually or colonoscopy every 10 years, while less than half employed DCBE, sigmoidoscopy and CT colonography.

**Conclusions:**

Although awareness of CRC screening in this study is high, its performance is very low and highly variable in form in our region. There is a need to improve the practice of CRC screening through sensitising of medical practitioners to the need for screening, increase knowledge with regard to the relative merits of available methodologies for screening/surveillance of CRC and provide all necessary diagnostic resources and possible formulation of effective local guidelines.

## Introduction

Colorectal carcinoma (CRC) is an important malignancy accounting for 9.4% of the global cancer burden in 2002 [1]. Previous reports[2, 3] had noted it to be rare among Africans. African fibre-rich diet and rarity of familial/hereditary colitis were thought to be protective. However, current reports indicate a rising global incidence [1]and a recent study showed an 81% increase in incidence over a period of two decades in Ibadan southwest Nigeria [4].Other reports indicate that it is now the commonest [5]gastrointestinal cancer in Lagos Nigeria with late presentations [6] and poor outcome. This rising prevalence of CRC has been attributed to improved cancer awareness and a shift towards Western diet. Screening of those at risk has been shown to be effective in reducing mortality from CRC while also cost-effective [7]. The evidence base for this exceeds that of other common malignancies that are widely screened for [8, 9], such as breast, cervical and prostrate cancers. Hence numerous guidelines exist for CRC screening including the recently published World Gastroenterology Organisation (WGO) [1] and the new Consensus Colorectal Cancer Guidelines by an American multidisciplinary panel of experts [10].

In the light of these developments we decided to evaluate the practice of CRC screening by medical doctors in Lagos, Southwest Nigeria. This report also sets out to ascertain the awareness and level of adherence to colorectal cancer screening guidelines by medical practitioners in Lagos State using the World Gastroenterology Organization Screening guidelines as a yardstick.

## Materials and Methods

This cross-sectional survey was conducted employing a self-administered questionnaire, which was distributed to over five hundred practising doctors in Lagos, Nigeria from September to November 2007. The distribution pattern aimed to recruit doctors from all levels of practice and the questionnaires were hand delivered to the doctors in public hospitals. For those in private practice, the forum for distribution was their monthly Medical Practitioners’ meeting. The data obtained from the questionnaire included biographical data, duration of practice, practice type, the doctor’s knowledge of the clinical features of colorectal carcinoma and their understanding and use of available screening techniques. The duration of practice was divided into three levels, short (≤5 yrs), medium (>5 yrs and ≤10 yrs) and long (>10 yrs). The awareness of the disease, type of screening criteria, modalities and frequency of screening were determined. The rate of screening for CRC was computed from methodologies used for screening by respondents and compared with screening rates for other malignancies. The statistical tests included Chi Square and student’s t test.

## Results

There were 300 respondents (60% of total). Most of the non-responders were elderly physicians who could not take out time to fill the questionnaires. Table 1 shows the features of the respondents.

**Table 1:** Basic demographics of respondents

Variables	Results
Mean age (SD)	33 (8) years
Sex (F: M)	1 : 2.4
Duration of practice	
Short duration (n, %)	190 (63%)
Medium duration (n, %)	43 (14%)
Long duration (n, %)	67 (23%)
Type of practice	
Teaching hospital (n, %)	197 (65.5%)
General hospital (n, %)	56 (18.5%)
Private practice (n, %)	47 (5.7%)

The scope of disease awareness of colorectal cancer among the respondents as well as criteria for screening for CRC in the population is shown in the Table 2.

**Table 2:** The scope of disease awareness of colorectal cancer amongst respondents

Variable	Number (freq)
Risk factors of CRC	
Dietary habits	277 (92.4%)
Familial	265 (88.8%)
Smoking	123 (41%)
Clinical manifestations	
Abdominal pain	204 (68%)
Abdominal mass	234 (77.5%)
Bloody mucoid stool	267 (88.7%)
Change in bowel habit	276 (92.3%)
Anemia	273 (91%)
Intestinal obstruction	261 (87.4%)
Knowledge of screening criteria	
Age of 40 years	256 (86.6%)
Family history of CRC	228 (76.6%)
Previous history of colonic polyps	240 (79.6%)
Inflammatory bowel disease	175 (64.7%)

**Figure 1:**
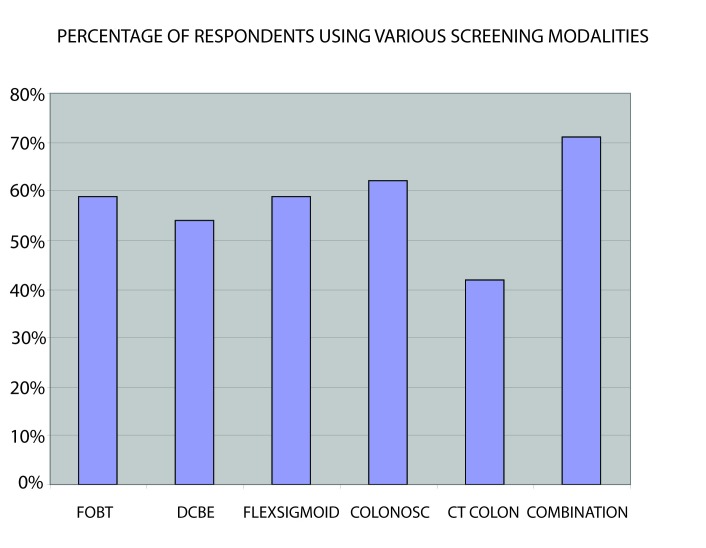
The screening methods. FOBT, Faecal occult blood test; DCBE, Double contrast barium enema; FLEX SIGMOID, Flexible Sigmoidoscopy; COLONOSC, Colonoscopy; CT COLON, CT Colonography; COMBINATION, Combination of 2 screening methods (Faecal occult blood test with double contrast barium enema or Sigmoidoscopy).

**Figure 2:**
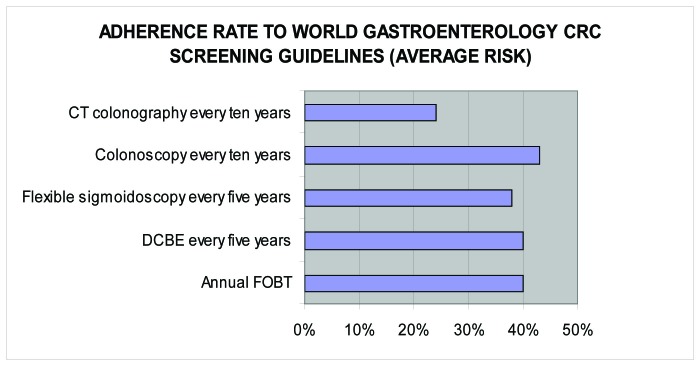
Adherence to WGO standard.

**Figure 3:**
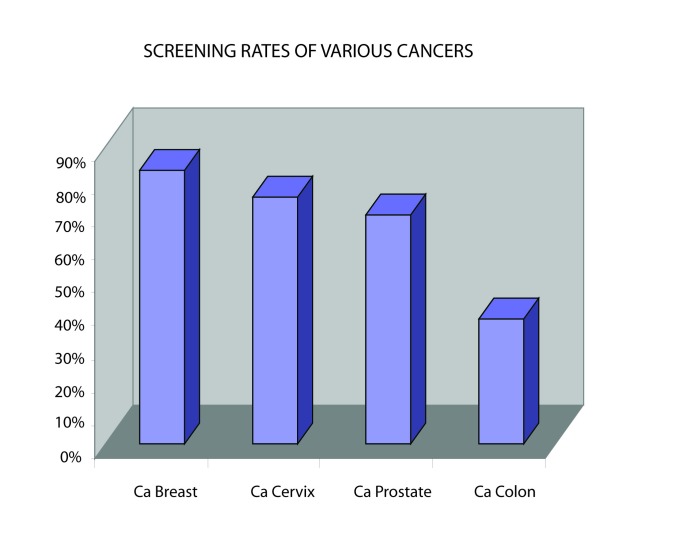
Screening rate for various malignancies encountered by respondents in their practice.

Screening for CRC was deemed worthwhile by 265 (87.8%). However, a small number, 21 (5%), was of the opinion that screening for CRC was not worthwhile while the others did not respond. Reasons given by those 246 (82%) who considered screening for CRC worthwhile included increasing prevalence, importance of early detection, impact of changing life style/westernisation and reduction of mortality/complications arising from the earlier detection. (Fig. 1-3).

## Discussion

Colorectal cancer is a notable cause of cancer death and is reported to be the second- most common cause of cancer-related deaths in North America [11]. It accounts for 10-50% of all GI malignancies in Nigeria [12-16] and present at advance stage associated with poor outcomes [6, 16, 17].This stage presentation of CRC in Nigeria is similar to that in other African countries [18, 19] and is in keeping with a predominantly unscreened population as has been reported in other parts of the world [20]. This survey that employed the recent WGO screening guideline on CRC has demonstrated a high degree of awareness of CRC, as well as indications and techniques appropriate for screening among medical practitioners in Lagos, Nigeria.

The cut off age of 45 years for screening for CRC was employed in the questionnaire because of recent reports from Nigeria that indicated that the age-related incidence of CRC in Nigerians was lower (almost by a decade**)** than that reported from developing countries [13, 15, 16].

However, utilization of the various screening tools by the respondents is below average and equally in the follow-up of patients at risk of CRC (surveillance), barely a quarter of them adhere to the guideline recommendation in terms of frequency of the screening. This low level of CRC screening may relate to diagnostic resource constraint as well as low awareness on screening among the populace. Previous studies [7] had shown that the most common reason why patients were not screened was that it was never recommended by their doctor.

The most commonly employed screening tool, however, is combination of different modalities followed by optical colonoscopy, annual FOBT and sigmoidoscopy. Not surprisingly, the screening technique least utilized in this report was CT colonography as this is a fairly recent screening modality for CRC. Though colonoscopy has proven to be the gold standard in CRC screening, it is not readily available in this part of the world due to cost and dearth of experienced endoscopists. The preference for combination of different technique (annual FOBT with sigmoidoscopy or DCBE) in this report is in line with a report from South Africa[21] that revealed this approach improved the yield over a single modality and was cost effective.

Across the globe and where CRC is common, experts attest to the need for CRC screening in view of the increasing risk due to changing life style, impact of early disease detection and intervention in improving disease outcome. However, the low level of CRC screening documented in this study in spite of its benefit is unfortunate as those at risk are not being screened since previous studies [7] had shown that the most common reasons why patients were not screened was that it was never recommended by their doctor.

The adherence rate for CRC screening in this study is below the 50% [8, 9] reported in the United States of America. In comparison with screening rates for other common cancers (breast, prostrate and cervical cancers) by the respondents, CRC was the lowest. This is also similar to previous reports from the Western countries [8, 9]. Higher screening rates for these cancers may relate to greater awareness by the public, as currently there exist enlightenment campaigns in the mass media for some like breast cancer.

There is a need to formulate local screening guidelines taking cognisance of resource availability in line with trends in other regions [10, 22] and use the mass media and other means to promote its use.

In conclusion, although awareness of CRC screening is high, its practice and use of the various techniques are very low in our local practice. Local guidelines in line with resource availability may need to be formulated and its use promoted using all media as currently exist for some cancers. Enlightenment of the populace particularly those at risk is also important. Proper screening for this all-important GI malignancy should be every medical practitioner’s business.
